# Cascaded Wx: A Novel Prognosis-Related Feature Selection Framework in Human Lung Adenocarcinoma Transcriptomes

**DOI:** 10.3389/fgene.2019.00662

**Published:** 2019-07-19

**Authors:** Bonggun Shin, Sungsoo Park, Ji Hyung Hong, Ho Jung An, Sang Hoon Chun, Kilsoo Kang, Young-Ho Ahn, Yoon Ho Ko, Keunsoo Kang

**Affiliations:** ^1^Department of Computer Science, Emory University, Atlanta, GA, United States; ^2^Deargen, Inc., Daejeon, South Korea; ^3^Division of Oncology, Department of Internal Medicine, College of Medicine, The Catholic University of Korea, Seoul, South Korea; ^4^Department of Molecular Medicine and Tissue Injury Defense Research Center, Ewha Womans University College of Medicine, Seoul, South Korea; ^5^Cancer Research Institute, College of Medicine, The Catholic University of Korea, Seoul, South Korea; ^6^Department of Microbiology, College of Natural Sciences, Dankook University, Cheonan, South Korea

**Keywords:** nonsmall cell lung cancer, cascaded Wx, CWx, feature selection, prognosis, machine learning, gene expression

## Abstract

Artificial neural network-based analysis has recently been used to predict clinical outcomes in patients with solid cancers, including lung cancer. However, the majority of algorithms were not originally developed to identify genes associated with patients’ prognoses. To address this issue, we developed a novel prognosis-related feature selection framework called Cascaded Wx (CWx). The CWx framework ranks features according to the survival of a given cohort by training neural networks with three different high- and low-risk groups in a cascaded fashion. We showed that this approach accurately identified features that best identify the patients’ prognoses, compared to other feature selection algorithms, including the Cox proportional hazards and Coxnet models, when applied to The Cancer Genome Atlas lung adenocarcinoma (LUAD) transcriptome data. The prognostic potential of the top 100 genes identified by CWx outperformed or was comparable to those identified by the other methods as assessed by the concordance index (*c*-index). In addition, the top 100 genes identified by CWx were found to be associated with the Wnt signaling pathway, providing biologically relevant evidence for the value of these genes in predicting the prognosis of patients with LUAD. Further analyses of other cancer types showed that the genes identified by CWx had the highest prognostic values according to the *c*-index. Collectively, the CWx framework will potentially be of great use to prognosis-related biomarker discoveries in a variety of diseases.

## Introduction

Lung cancer is the most commonly diagnosed cancer and the second most common cause of cancer-related deaths worldwide (Bray et al., 2018). Most lung cancer cases are nonsmall cell lung cancer (NSCLC), and lung adenocarcinoma (LUAD) accounts for more than 50% of all NSCLCs. Recently, survival rates for LUAD patients have been greatly improved with the development of improved treatment approaches, including surgical or radiation techniques, and the introduction of targeted therapies and immunotherapies tailored to the molecular or immunologic characteristics of tumors. However, the survival rate is still only about 50% for potentially curatively resected LUAD (Xia et al., 2017). To optimize clinical intervention, it is important to identify which patients have poor prognoses. The prediction of prognosis requires an extensive knowledge of various aspects of cancer biology and an understanding of relevant clinical information such as TNM stage, histology, and genetic mutations (Greaves et al., 2011). Among the clinical features, TNM staging is the most successful clinical parameter in practice and is widely used to predict patients’ prognoses. However, this staging method still has room for improvement in the era of genomic sequencing, where abnormalities in multiple genes can be detected simultaneously (Roukos, 2010). Among the various genome-wide applications, the gene expression signature is the most promising approach to the prediction of clinical outcomes (van’t Veer et al., 2002; Ramaswamy and Perou, 2003; Chibon, 2013), as a suite of expressed genes reflects the identity of a given cell population. Several gene expression-based clinical applications such as MammaPrint (Wittner et al., 2008) and Oncotype DX (Carlson and Roth, 2013) are being used in clinical practice. These applications predict patients’ prognoses and drug and/or chemotherapy responsiveness by examining the expression levels of a defined gene set. Therefore, the identification of a particular gene set associated with clinical findings is crucial in many disease research studies.

Recent technological advancements in clinical genome sequencing using next-generation sequencing (NGS) technologies provide opportunities to understand the relationships between gene expression and tumor phenotypes (Koboldt et al., 2013). For example, several studies classify NSCLC patients into subgroups with differing clinical outcomes using gene signatures (Chen et al., 2007; Skrzypski et al., 2008; Boutros et al., 2009; Xie et al., 2011). However, the results of such studies have been unsatisfactory in terms of discrepancies between identified gene signatures. The possible reasons for the inconsistent results among the studies include the use of small samples compared to the number of genes (high-dimensional data), the use of different platforms, and the problems with feature preprocessing steps. In addition, there are no robust methods for analyzing such high-dimensional data effectively.

Machine learning (ML) algorithms can be a useful approach to the analysis of high volumes of data if a model is well constructed with high-quality input data for training. Numerous variations of the original ML algorithms have been developed and applied to a variety of problems (Litjens et al., 2017; Park et al., 2017; Zhang et al., 2017; Esteva et al., 2019). In molecular biology, NGS technologies, which revolutionized the profiling approach by sequencing huge numbers of given short DNA fragments, have been generating enormous amounts of data these days (Goodwin et al., 2016). Because of this, there is an urgent need to develop ML-based algorithms that can effectively analyze such high volumes of genomic data. Support vector machines (SVM; Chang and Lin, 2011), *k*-nearest neighbors (Cover and Hart, 1967), multilayer perceptrons (Mateos et al., 2002), decision trees (Chou et al., 2013), random forest (RF; Zhang et al., 2016) algorithms, logistic regression, and gradient boosting machines (Mall et al., 2018) are ML algorithms that are frequently used to analyze big data. However, these methods were not originally designed to extract prognostic features from patients’ data. Recently, several ML-based algorithms have been proposed to select a subset of key features (genes) for classification (Anaissi et al., 2013; Yao et al., 2015; Freres et al., 2016) or to identify prognostic features (Wenric and Shemirani, 2018) from high-throughput molecular profiling data. There is still room for improvement, however, as new deep learning algorithms continue to emerge in the field of ML (Devlin et al., 2018; Peters et al., 2018).

To effectively analyze multidimensional datasets, dimension-reduction algorithms such as feature selection are often required. Principal component analysis (PCA; Jolliffe, 2011), nonnegative matrix factorization (Lee and Seung, 2001), kernel PCA (Mika et al., 1999b), graph-based kernel PCA, linear discriminant analysis (Mika et al., 1999a), and generalized discriminant analysis (Baudat and Anouar, 2000) are algorithms that are widely applied to high-dimensional biomedical datasets. In addition to these approaches, several studies recently used artificial neural networks to predict clinical outcomes in lung cancer patients (Jefferson et al., 1997; Xie et al., 2014; Hart et al., 2018). However, these approaches do not fully take into account available information such as high-throughput profiling data (e.g., transcriptomes) and/or clinical information for feature selection. To address these problems, we developed a novel feature selection framework called Cascaded Wx (CWx) to enhance the efficiency of feature selection and the accuracy of prediction for given patients’ prognosis. Our analyses revealed that the CWx framework selected more prognosis-related features than algorithms in categories such as similarity-based, sparse learning-based, ML-based, and statistical-based models, highlighting the potential value of our proposed framework for biomedical data.

## Materials and Methods

### Data Acquisition

Gene expression data (mRNASeq) from 507 LUAD, 495 lung squamous cell carcinoma (LUSC), 1,091 breast invasive carcinoma (BRCA), 405 bladder urothelial carcinoma (BLCA), and 97 rectum adenocarcinoma (READ) patients were obtained from The Cancer Genome Atlas (TCGA) *via* the firehose browser (https://gdac.broadinstitute.org/). The data were generated by the Illumina HiSeq instrument (labeled as illuminahiseqrnaseqv2-RSEMgenesnormalized). We extracted gene features (*X*), survival values (*S*), and censoring information (*C*), which can be formally represented as *X* ∈ *R^n^* ^×^ *^d^*, *S* ∈ *R^n^*, and *C* ∈ *R^n^*, respectively; *n* is the number of patients and *d* is the feature dimensionality. If *C_i_* = 0 (uncensored patients), the survival time interval represents the time between the start of observing the patient status and the event (date of death) time. If a patient datum is right censored (*C_i_* = 1), the survival time interval represents the time elapsed between the start of observing the patient status and the end of the study. These data should not be included when training a survival model, because they can be regarded as missing data. More details are discussed in the survival evaluation model section. Of the 507 LUAD patients, there were 183 uncensored (death event occurred) samples and 324 right-censored samples. For other cancer types, there were 283, 940, 227, and 79 right-censored samples for LUSC, BRCA, BLCA, and READ, respectively. Each sample contained read counts (expression levels) of 20,501 genes. These count-based values were abundant for a few specific transcripts (highly expressed genes), a factor that prevents a model from finding a good pattern. To mitigate this problem, we used a log transformation:

$$X_{ij}^{new}=log_{2}(X_{ij}+1),$$

for *i* ∈ *n* and *j* ∈ *d*. A constant, 1, was added to the read count value of each gene before applying the logarithm function to avoid problems with zeros. Min-max normalization was then applied to the log-transformed data.

### Development of a Novel Prognosis-Related Feature Selection Framework: CWx

The proposed method was based on the Wx algorithm (Park et al., 2017), which identifies key genes discriminating between different groups, such as normal vs. cancer, based on transcriptome (RNAseq) data. The top features were selected using the following discriminating power (DP) equation:

$$DP_{j}=\left|W_{normal}{\hat{X}}_{j,normal}-W_{cancer}{\hat{X}}_{j,cancer}\right|$$

**Figure 1:**
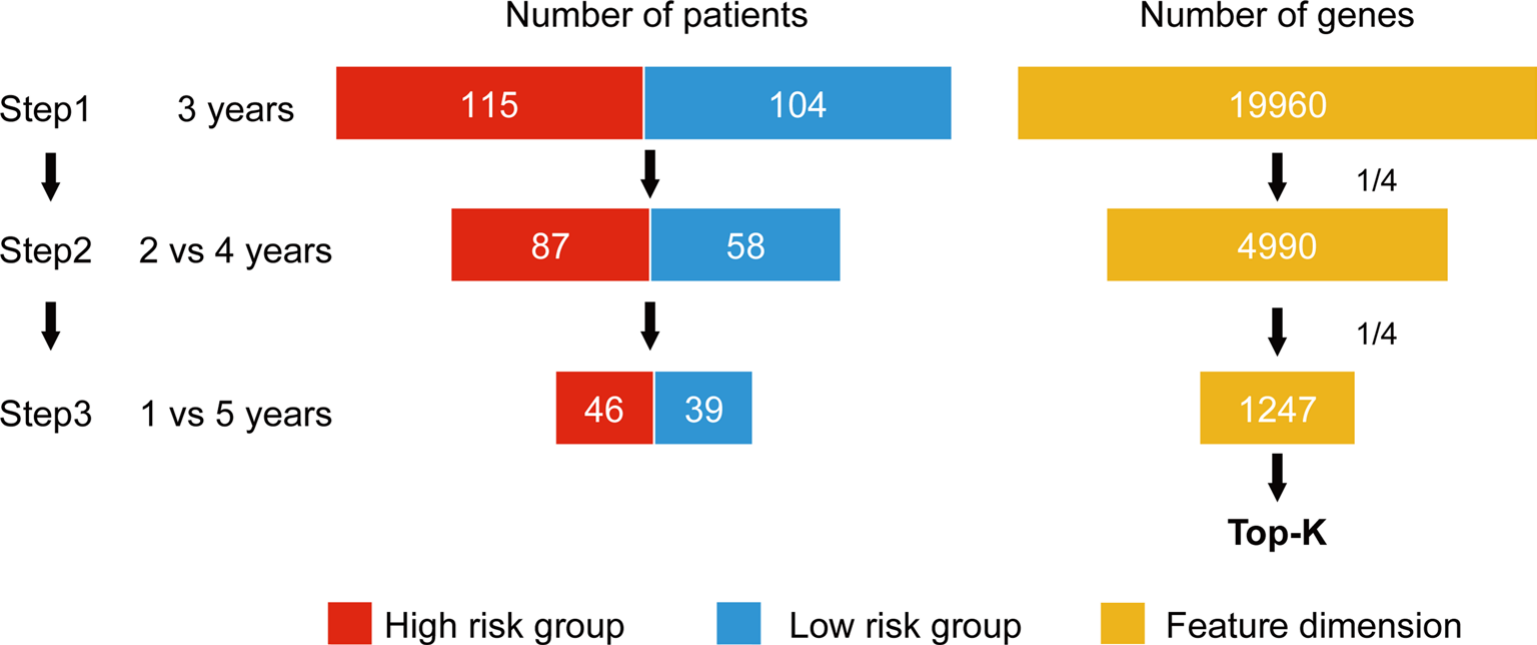
An example of CWx’s feature selection procedure. Input samples (patients) are reduced through three cascaded steps using different criteria. Three-year, 2 versus 4-year, and 1- versus 5-year cutoffs for categorizing samples into either high- or low-risk groups are used at first, second, and third steps, respectively. Input features (genes) are also reduced by a quarter in each step. Finally, the prognostic potential of features can be estimated according to the weights calculated from the trained neural network.

*W_normal_* and *W_cancer_* represent trained weights linked to the normal and cancer output of the softmax, respectively. ${\hat{X}}_{j,normal}$ is the average of the feature *j* for the class, “normal,” and likewise, ${\hat{X}}_{j,cancer}$ is the average of feature *j* for the class, “cancer.” As this method was designed to be applied to a classification problem, we cannot apply it to the survival analysis as is. Therefore, in this study, we propose a novel prognosis-related feature selection algorithm, CWx, which identifies prognosis-associated features (genes) from a large amount of patient transcriptome data, together with clinical information. The basic concept of the CWx algorithm is to improve learning performance by reducing the number of samples (patients) and the number of features (genes) over the course of three steps (**Figure 1**). In the first step, patients were divided into high- and low-risk cohorts according to whether they have survived for 3 years. For example, 115 deceased patients within 3 years in a training set formed one group (28.4%; high risk), whereas 104 patients who lived more than 3 years formed another group (25.7%; low risk). The remaining patients (186, 45.9%) were right censored, meaning that there was no information as to whether these patients were deceased within 3 years. These right-censored patients were excluded in the training stage. The second and third steps are similar to the first step with different cutoffs (2 versus 4 years and 1 versus 5 years, respectively). As with the strategy of reducing the number of samples, the number of features (genes) was also reduced by a quarter in each step. One quarter of the features was selected according to the importance determined by our previous Wx feature selection algorithm (Park et al., 2017). A total of 19,960 genes were used as input features after removing genes with no variance. The final output is a set of genes ranked by prognostic weights, estimated in a manner similar to the Wx algorithm (Park et al., 2017). The code for the CWx algorithm is available on the GitHub website (https://github.com/deargen/DearCascadedWx).

### Survival Evaluation Model

The survival evaluation model used in this paper is the Faraggi–Simon method (Faraggi and Simon, 1995), which is a nonlinear proportional hazards model. This model incorporates a negative log-partial likelihood as a cost function, which can be represented as follows:

$$\log L(\theta )=\sum_{i:C_{i=0}}\left[f(X_{i},\theta )-\log\sum_{j:\in S_{j}\geq S_{i}}e^{f}(x_{j},\theta )\right],$$

where *f*(*X_k_*, θ) is a log-hazard rate parameterized by the weights of the network, θ. In this study, a single-layer feed-forward neural network was used as a nonlinear function, *f*. We used 100 epochs for training and 20 epochs for early stopping, with the adaptive moment estimation optimizer (Kingma and Ba, 2014). The batch size was equal to the number of whole training data samples, because the negative log likelihood cost function calculates the likelihood of the whole dataset at once. We selected the best learning rate based on the best concordance index (*c*-index) on the validation set, which was 20% of the training dataset.

### Evaluation Metrics

A stratified fivefold cross-validation method was used to check general model behaviors. For each selected test subset, the other four subsets were used as the training set. Therefore, the overall result was the average of the five subresults. The performance of the algorithms was evaluated using the log-rank test (Altman, 1990) as well as Harrell’s *c*-index, a nonparametric statistic that measures concordance between predicted risk and actual survival (Harrell et al., 1982).

### Merging Selected Features

Because we used the fivefold cross-validation method, which produces five different lists (sublists) of feature (gene) rankings, these gene lists were merged to generate a representative gene list for each method. The summation of a given set of five sublists was conducted as formulated below:

$$Gene\;Ranking\;Point_{j}=\sum_{k=1}^{5}\left[N-R_{jk}\right],$$

where *j* ∈ *d*, *N* is the total number of features and *R_jk_* is the ranking of gene *j* in *k*th fold. The final representative gene list was determined by sorting the gene ranking points in descending order.

### Feature Selection Methods

Feature selection is a common approach in computer science to reduce dimensionality. This approach is extremely useful when it comes to genomic datasets, which typically contain more than 20,000 features (genes). We used the following feature selection methods for comparisons.

#### Cox Proportional Hazards (CoxPH)

The CoxPH model (Cox, 1972) is a regression model designed for survival analysis with respect to patients’ features. This model is one of the most widely used methods in survival analysis. The Cox model is formulated as the risk function:

$$\eta (t,X)=\eta_{0}(t)\cdot e^{\beta^{T}X},$$

where the risk of an event at the survival time *t* ⋅ η_0_(*t*) is the baseline hazard, β ∈ *R^p^* is the coefficient to be learned, a measure of the impact of features, and X ∈ *R^p^* is the input feature.

#### Coxnet

We used Coxnet, a Cox regression model with an Elastic-Net penalty (Zou and Hastie, 2005), as another comparative method. Elastic-Net was chosen as one of the algorithms as it effectively incorporates L1 and L2 penalties into its cost function to select a parsimonious feature set. Although the algorithm automatically sets the number of features selected, it provides a good baseline for survival analysis with a succinct set of features. We use the Python package glmnet (Simon et al., 2011) as an Elastic-Net implementation. The regularization term of Elastic-Net is represented as follows:

$$P_{\alpha }(\lambda ,\beta )=\lambda \left(\alpha \sum_{i=1}^{p}\left|\beta_{i}\right|+\frac{1}{2}(1-\alpha )\sum_{i=1}^{p}\beta_{i}^{2}\right)$$

The parameter λ controls the level of the regularization, and α weights LASSO higher when it approaches one, and Ridge regression when it approaches zero. This yields the benefit of discrete feature selection from LASSO and the ability to handle correlated features from Ridge regression. The Python package lifelines (version 0.14.1) was used.

#### ML-Based Models

One of the major categories of feature selection methods is based on generic ML models, such as RF (Breiman, 2001), SVM (Cortes and Vapnik, 1995), connection weight (Olden et al., 2004), and extreme gradient boosting (XGBoost; Chen and Guestrin, 2016). The feature selection process of these methods is first applying the model to a problem and then analyzing the trained model to select a salient group of features. For these methods, the Python package scikit-learn (version 0.19.1) was used.

#### Similarity-Based Feature Selection

One group of feature selection methods is designed to preserve sample similarity. These approaches implicitly select partial features that maintain similarity. However, the similarity-based feature selection algorithms can be subcategorized, as they have different goals. ReliefF (Kononenko, 1994) and the Fisher score (Duda et al., 2012) focus on separability, whereas Trace ratio (Nie et al., 2008) targets locality. The Python package skfeature-chappers (version 1.0.3) was used to run the algorithms.

#### Sparse Learning-Based Feature Selection

Like Elastic-Net, sparse learning-based feature selection methods incorporate both L1 and L2 regularizers. The difference between this group and Coxnet is the cost function. The cost function of Coxnet is the proportional hazards function, whereas the sparse learning-based feature selection methods are based on the classification problem. There are several variants of these methods with minor modifications, such as robust feature selection (RFS; Nie et al., 2008) and LLL21 (Liu et al., 2009). The RFS and LLL21 algorithms were compared to the proposed method. The Python package skfeature-chappers (version 1.0.3) was used.

#### Statistical-Based Feature Selection

The last group we used for comparison is based on statistics, where each feature is selected according to various standardized test statistics. For example, Fscore, Tscore, and DESeq2 use analysis of variance (ANOVA) of scores, *t*-scores, and a negative binomial distribution (Love et al., 2014), respectively. In this paper, we only included Fscore and DESeq2 results, as Tscore identified only a limited number of genes. The Python package skfeature-chappers (version 1.0.3) and the R package DESeq2 (version 1.22.2) were used, respectively.

### Overview of the Evaluation Pipeline

**Figure 2:**
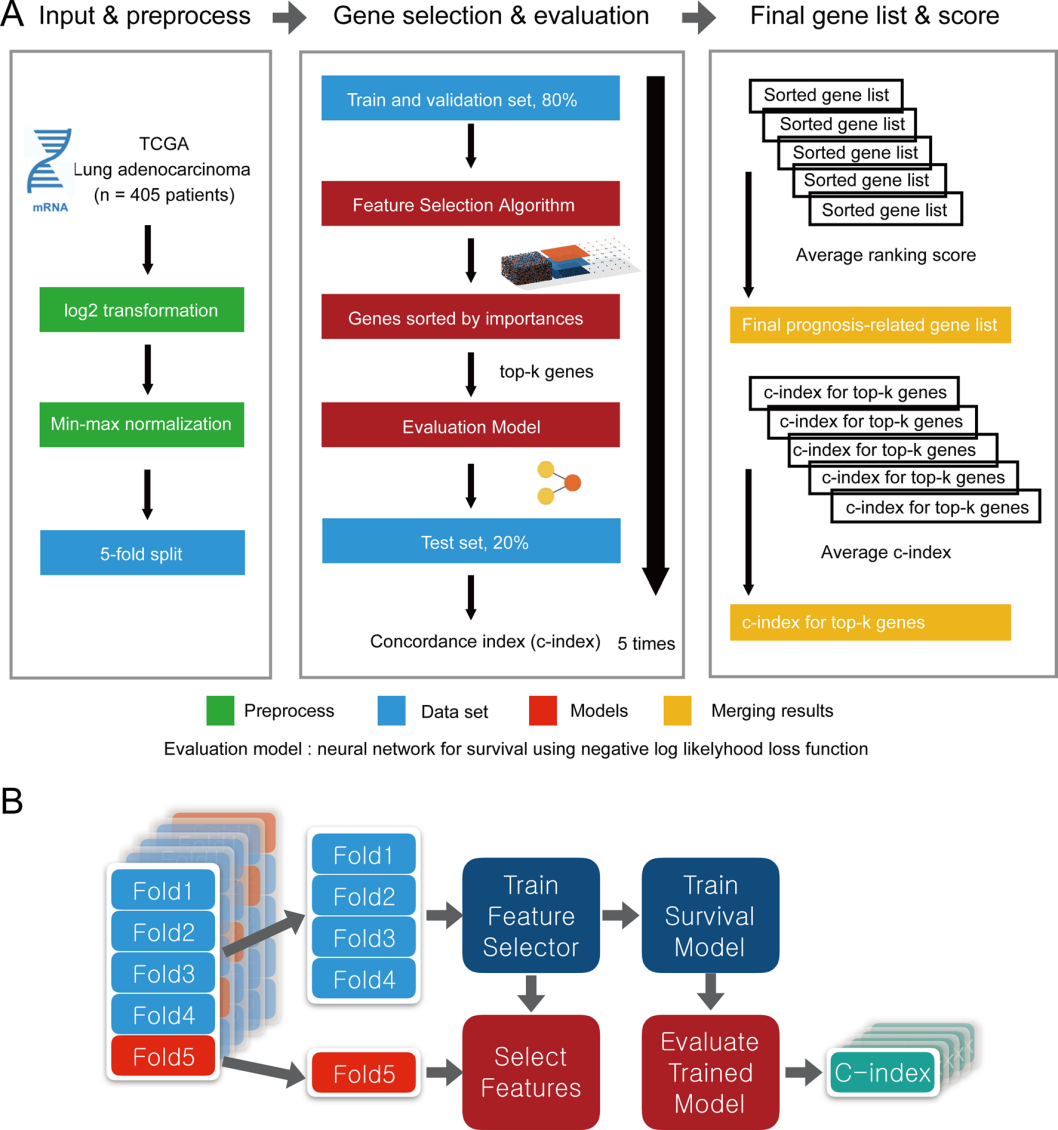
Overview of the evaluation pipeline. **(A)** **Overview of data preprocessing, normalization, and evaluation pipeline.** **(B)** **Process of fivefold cross-validation for training models and evaluating trained models. c-index was used as the performance measurement.**

To evaluate the performance of CWx with other feature selection algorithms, we used transcriptome data obtained from TCGA. Log transformation, a widely used method to reduce skewness, was applied, and then the data were further normalized using min-max normalization (**Figure 2**). The data were divided into five subgroups without intersection for fivefold cross-validation. The fivefold cross-validation was performed using the top genes identified by a given feature selection algorithm, and performance was evaluated by averaging the reported performance measures (*c*-index; **Figure 2A**). Because the number of samples was small, we performed fivefold cross-validation for both a feature selector and a survival model to avoid (un)lucky peaks, as shown in **Figure 2**. For each split, we held out one subset as a test dataset (light reds in **Figure 2B**), whereas other remaining subsets (light blues in **Figure 2B**) were used for training both a feature selector and a survival model. For the survival model, we used conventional negative log likelihood. The Kaplan–Meier survival plot with the log-rank test and *c*-index was also used to evaluate the genes identified by each algorithm.

## Results

### Comparison of Feature Selection Algorithms for Prognosis Prediction

**Figure 3:**
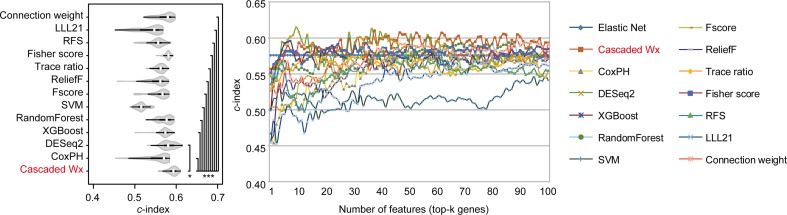
Comparison of feature selection algorithms with cumulative top *k* genes. Violin plot shows the *c*-indexes of the top genes (cumulative from 1 to 100 in lung adenocarcinoma (LUAD) samples; *n* = 100) identified by each algorithm (left). White circles indicate the medians; box limits inside the polygons indicate the 25th and 75th percentiles as determined by R software; whiskers extend 1.5 times the interquartile range from the 25th and 75th percentiles; polygons represent density estimates of data and extend to extreme values. Asterisks (*P < 0.05 and ***P < 0.001) indicate the results of one-way ANOVA (*P* < 0.0001) with *post hoc* test (pairwise *t* test with Bonferroni–Holm correction). *x*- and *y*-axes indicate the number of cumulative top genes and *c*-index, respectively (right).

To compare the prognosis-related gene selection performance of CWx with the current state-of-the-art feature selection algorithms, we used TCGA transcriptome data (expression levels of 20,501 genes) of LUAD (*n* = 507) together with clinical information. The dataset contained 324 censored and 183 events (deceased). Patients were categorized into either the high-risk group or the low-risk survival group according to a 3-year survival outcome (censored or deceased), making this a binary classification problem. We compared the proposed algorithm, CWx, to the following supervised feature selection algorithms from five different categories: i) ML-based models: RF (Breiman, 2001), SVM (Cortes and Vapnik, 1995), XGBoost (Chen and Guestrin, 2016), and connection weight (Olden et al., 2004); ii) similarity-based models: Fisher score (Duda et al., 2012), ReliefF (Kononenko, 1994), and Trace ratio (Nie et al., 2008); iii) sparse learning-based models: multitask feature learning *via* efficient l2,1-norm minimization (LLL21; Liu et al., 2009) and RFS (Nie et al., 2010); iv) statistical-based models: Fscore and DESeq2 (Love et al., 2014); and v) others: CoxPH (Cox, 1972). The information theoretical-based algorithms such as max-relevance min-redundancy (Peng et al., 2005), conditional mutual info maximization (Fleuret, 2004), and conditional infomax feature extraction (Lin and Tang, 2006) were excluded for evaluation due to the small numbers of features identified by the algorithms (<100 features). These algorithms calculate a score for each given feature, so the performance of each cancer prognosis prediction can be estimated by comparing the highest-scoring features selected by each algorithm. The Python package “skfeature-chappers” (version 1.0.3; https://pypi.org/project/skfeature-chappers/) was used for the feature selection algorithms, and the top 100 features, as ranked by the feature importance score (or feature coefficient) calculated by each algorithm, were used for the comparisons. The importance assigned to features by ML algorithms, which were not originally intended for feature selection, was determined by estimating the importance for XGBoost and RF and by assessing a coefficient for SVM. The “xgboost” Python package (version 0.71) was used to apply the XGBclassifer” function, and the “scikit-learn” Python package (version 0.19.1) was used to apply the “SVC” (SVM) and “RandomForestClassifer” functions. We also compared CWx to CoxPH and Coxnet as baseline methods for prognosis prediction. Feature selection criteria for CoxPH and Coxnet were *P* value and beta coefficients, respectively. Because Coxnet produced less than 50 genes, we could not calculate the *c*-index of the top 100 genes for Coxnet. We therefore used the results from Coxnet, with all known genes used after model learning, as the baseline performance for comparison. The results indicated that CWx was superior to the other methods in terms of *c*-index when comparing the top genes (cumulative) from 1 to 100 in LUAD samples (**Figure 3** and **Table 1**). We also evaluated the algorithms with the log-rank test using the top *k* genes. CWx showed the most significant *P* value (9.6E-8) when the top 5 genes were used followed by RFS, Fisher score, and Fscore (**Table 2**). However, RFS was the best performer in the other three comparisons using top 10, top 50, and top 100 genes. Next, we further evaluated the algorithms’ performance with different cancer datasets such as LUSC, BRCA, BLCA, and READ. The results showed that CWx was the best performer in BRCA and READ. In contrast, Trace ratio and RF were the top performers in LUSC and BLCA, respectively, followed by CWx in both cancer types (**Figure S1** and **Table 3**). Overall, the comparisons demonstrated that the CWx framework was superior in identifying prognosis-related genes in cancer transcriptome data.

**Table 1:** Summary of *c*-indexes for lung adenocarcinoma (LUAD) patients using top genes.

	Top 5	Top 10	Top 50	Top 100
CWx	0.5670	0.5786	**0.5971**	**0.5932**
CoxPH	0.5077	0.5072	0.5709	0.5709
DESeq2	**0.5943**	**0.6148**	0.5813	0.5727
XGBoost	**0.5833**	0.5687	0.5719	0.5849
RF	0.5541	0.5593	0.5752	0.5741
SVM	0.5121	0.5230	0.5054	0.5415
Fscore	0.4981	0.5161	0.5641	0.5805
ReliefF	0.5215	0.5377	0.5569	0.5704
Trace ratio	0.5502	0.5624	0.5616	0.5539
Fisher score	0.5756	**0.5814**	**0.5903**	0.5742
RFS	0.5639	0.5111	0.5650	0.5546
LLL21	0.4927	0.4915	0.5470	0.5614
Connection weight	0.5319	0.5424	0.5882	**0.5917**

The red and bolded texts represent the first and second highest scores in each category, respectively.

**Table 2:** Summary of log-rank *p* values for 3-year survival of LUAD patients using top genes.

	Top 5	Top 10	Top 50	Top 100
CWx	**9.60E-08**	**2.10E-09**	1.20E-12	9.00E-27
CoxPH	2.00E-02	1.60E-03	7.60E-14	7.10E-19
DESeq2	9.00E-03	2.00E-04	1.00E-14	7.30E-20
XGBoost	2.90E-04	1.70E-05	4.40E-13	1.70E-17
RF	3.40E-03	3.00E-04	1.10E-15	3.00E-20
SVM	7.30E-04	2.00E-05	**1.20E-27**	**5.80E-41**
Fscore	1.20E-05	1.10E-08	5.10E-19	1.60E-25
ReliefF	1.40E-04	2.20E-06	3.90E-18	2.90E-27
Trace ratio	1.10E-05	1.90E-08	1.30E-18	5.10E-25
Fisher score	1.20E-05	1.10E-08	5.10E-19	1.60E-25
RFS	**3.50E-06**	**1.20E-11**	**4.40E-40**	**4.20E-50**
LLL21	1.50E-02	1.20E-02	3.30E-13	2.20E-16
Connection weight	1.20E-02	2.00E-03	1.50E-14	1.20E-27

The red and bolded texts represent the first and second highest scores in each category, respectively.

**Table 3:** Average *c*-index (top genes range from 1 to 100; *n* = 100) of five different cancer cohorts.

	LUAD	LUSC	BRCA	BLCA	READ
CWx	**0.5918**	**0.5558**	**0.6331**	**0.6060**	**0.7482**
CoxPH	0.5553	0.5476	0.5513	0.5914	0.6401
DESeq2	**0.5824**	0.5258	0.5465	0.5398	0.5582
XGBoost	0.5736	0.5223	0.6099	0.5976	0.6379
RF	0.5735	0.5546	0.5774	**0.6114**	**0.7392**
SVM	0.5164	0.5306	0.5520	0.5378	0.4478
Fscore	0.5602	0.5477	0.5702	0.5552	0.5316
ReliefF	0.5544	0.5423	0.5820	0.5641	0.5501
Trace ratio	0.5622	**0.5611**	0.6100	0.5732	0.6338
Fisher score	0.5802	0.5433	**0.6187**	0.6058	0.7099
RFS	0.5541	0.5453	0.5794	0.5676	0.5308
LLL21	0.5358	0.5324	0.5198	0.5808	0.5956
Connection weight	0.5720	0.5482	0.5350	0.6040	0.6353

The red and bolded texts represent the first and second highest scores in each category, respectively.

### Functional Analysis of Prognosis-Related Genes

**Figure 4:**
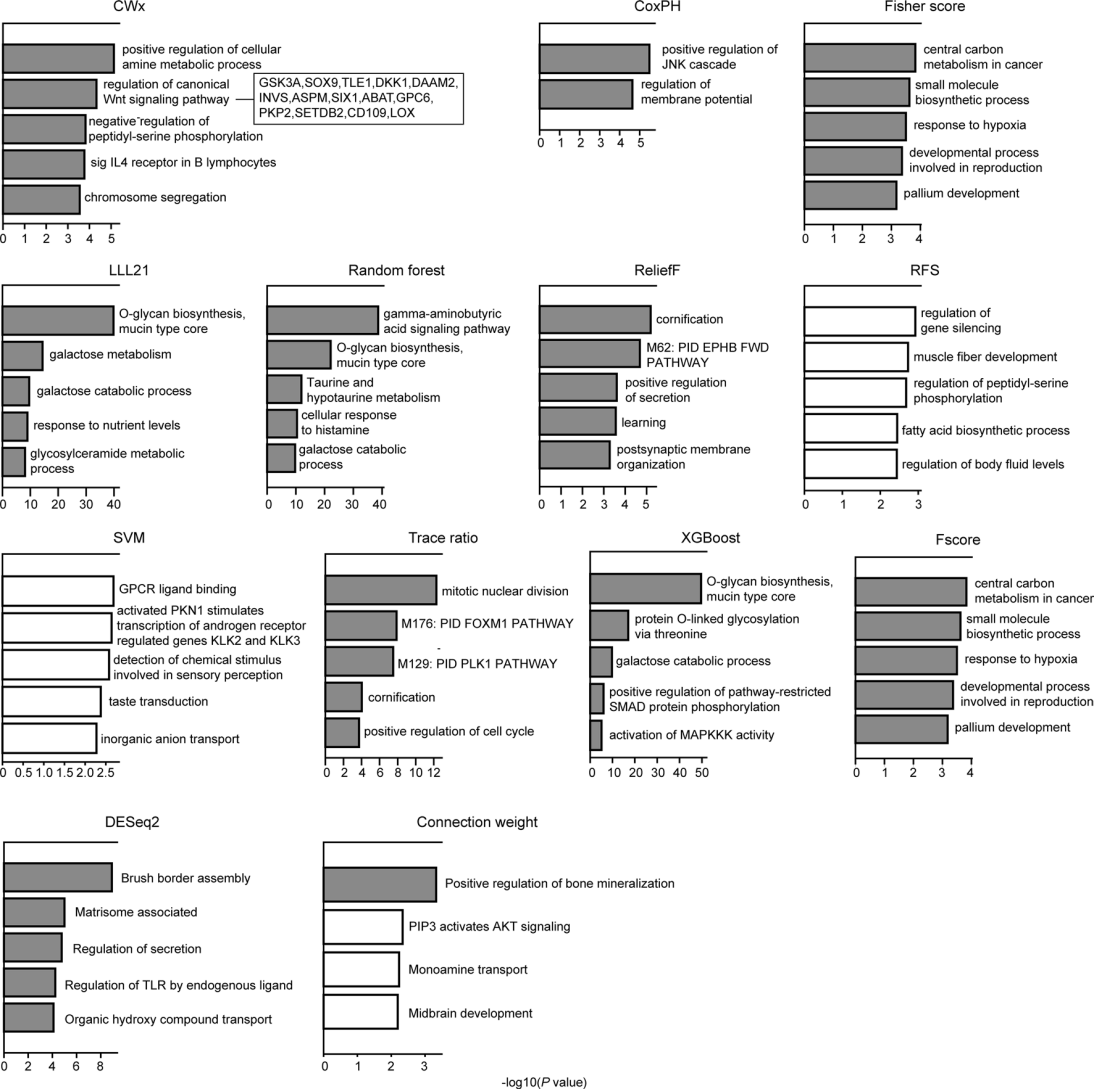
Gene ontology (GO) analysis of top 100 genes. GO analysis was performed using Metascape (http://metascape.org/gp/index.html) with top 100 genes (default parameters were used). The significance of a given GO term is represented by gray (significant) or white (nonsignificant) bars with a *P* cutoff value of 0.0001.

We compared the top 100 gene sets identified by each algorithm to ensure that there were core gene signatures in LUAD. Interestingly, there was little overlap even between the fivefolds (groups) that were used for cross-validation by each algorithm (**Figure S2**). DESeq2 showed the highest overlap (10.0%), whereas CWx had 2.4% of overlap observed between the fivefolds. XGBoost, RF, RFS, and connection weight showed no overlap between the fivefolds. The result is likely to be due to a relatively small number of samples for training and/or algorithmic differences. Next, we performed Gene Ontology (GO) analysis to identify the biological pathways associated with the top 100 genes. This analysis revealed that the gene set identified by CWx was associated with the Wnt signaling pathway (**Figure 4**), one of the key pathways regulating development, and closely associated with many cancers. The gene sets identified by the other algorithms were related to different pathways such as “positive regulation of JNK cascade” (CoxPH), “central carbon metabolism in cancer” (Fisher score and Fscore), “O-glycan biosynthesis, mucin type core” (LLL21, RF, and XGBoost), “mitotic nuclear division” (Trace ratio), “regulation of gene silencing” (RFS), and “GPCR ligand binding” (SVM). Differences between the gene sets identified by the different algorithms, and their associated biological pathways, need to be further investigated in future studies.

### Evaluation of the Cascaded Framework for the Prognosis Analysis of LUAD Patients

**Figure 5:**
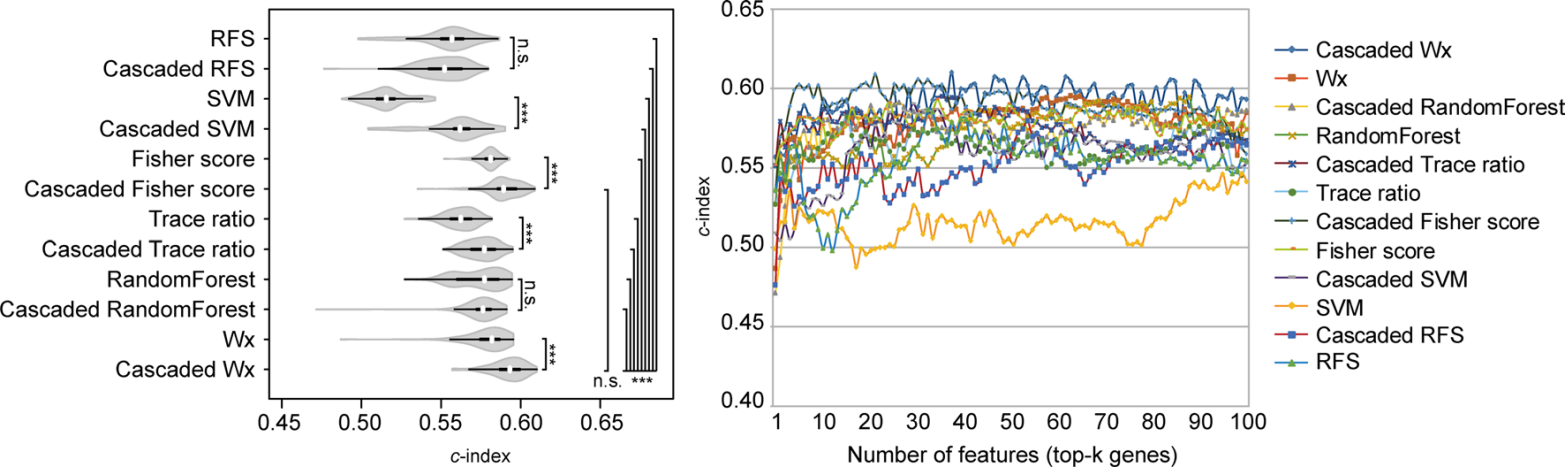
Evaluation of the cascaded framework. Violin plot shows the *c*-indexes of the top genes (cumulative from 1 to 100 in LUAD samples; *n* = 100) identified by each algorithm with or without the cascaded framework (left). White circles indicate the medians; box limits inside the polygons indicate the 25th and 75th percentiles as determined by R software; whiskers extend 1.5 times the interquartile range from the 25th and 75th percentiles; polygons represent density estimates of data and extend to extreme values. Asterisks (*P < 0.05; ***P < 0.001; n.s., not significant) indicate the results of one-way ANOVA (*P* < 0.0001) with *post hoc* test (pairwise *t* test with Bonferroni–Holm correction). *x*- and *y*-axes indicate the number of cumulative top genes and *c*-index, respectively (right).

The above comparison was conducted by comparing the CWx framework to various ML algorithms, which do not incorporate the cascaded framework. We wondered whether the incorporation of the cascade framework could also improve the performance of the other ML algorithms for prognosis analysis. To this end, we applied the cascade framework to the Fisher score, RF, Trace ratio, SVM, and RFS algorithms and compared them to CWx. The evaluation revealed that the cascade framework significantly improved the feature selection performance for SVM, Fisher score, Trace ratio, and Wx (our previous feature selection algorithm) compared to the algorithms without the framework (**Figure 5**), although the CWx model still showed the best performance in terms of *c*-index. Interestingly, the cascaded framework failed to improve performance for both RFS and RF.

## Discussion

Lung cancer is one of the leading causes of cancer-related deaths worldwide. The identification of prognostic biomarkers is a primary goal of lung cancer studies. In this study, we developed a neural network-based prognosis-related feature selection framework to improve the performance of current prognosis prediction models. Our proposed CWx framework identifies prognosis-related features through a cascaded approach, as shown in **Figure 1**. Our evaluation using 507 TCGA LUAD transcriptomes revealed that the prognosis-related gene set identified by CWx either outperformed or matched the performance of the gene sets extracted by the other classifiers using a stratification of samples into low- and high-risk categories according to the *c*-index. This finding means that the prognosis-related gene set found by CWx is one of the best candidate gene sets to predict patients’ prognoses. This feature reduction framework is a very important technology in the era of NGS, in which expression values for tens of thousands of genes are routinely calculated.

The CWx framework was designed to select the optimal gene set associated with patients’ prognoses using the survival information of a given cohort and changing the separation criteria between high- and low-risk groups through a three-step cascade method. Therefore, the CWx algorithm can be applied to the identification of prognosis-related genes associated with a range of diseases, not only LUAD (**Figure S1**). In addition, CWx has a linear execution time to complete the feature selection steps depending on the number of samples. Some information theoretical-based feature selection algorithms take longer to finish the feature selection procedure. In contrast, one of the disadvantages of CWx is that it can only handle right-censored data within 3 years due to the binary classification of patients into either high- or low-risk groups. However, all of the supervised feature selection algorithms have this problem when applied to survival analysis. One possible solution to this issue is to select features directly from a given neural network training model using a negative log-likelihood cost function that can handle the whole sample for survival analysis.

One of the key pathways related to the prognosis of LUAD patients identified by the CWx framework was the Wnt signaling pathway. A recent study has shown that two distinct subpopulations of cells, one with high Wnt signaling activity and another forming a niche that provides the Wnt ligand, are activated in LUAD. In addition, *in vitro* and *in vivo* studies have suggested that Wnt responsiveness contributes to the survival of cancer cells and the maintenance of a stem cell-like niche cell phenotype (Tammela et al., 2017). Interestingly, several prognosis-related genes identified by the CWx framework have been previously reported in LUAD studies. For example, glycogen synthase kinase 3 is a central regulator of cellular metabolism, development, and growth and is frequently elevated in NSCLC, supporting tumor cell proliferation (Vincent et al., 2014). Several SRY-related HMG box (SOX) genes, such as SOX2, SOX4, SOX7, SOX9, SOX11, and SOX17, have been known to be expressed in the developing lung, and it has been suggested that they are involved in the abnormalities of lung morphogenesis and function. Of these SOX genes, SOX9 is frequently up-regulated in LUAD (Maeda et al., 2007). SOX9 affects the expression of the cell cycle regulators p21 and cyclin-dependent kinase 4 and thus contributes to an increase in lung cancer growth potential (Jiang et al., 2010). Transducin-like enhancer of split 1 (TLE1) is a transcriptional corepressor that interacts with a variety of DNA-binding transcription factors and has been implicated in many signaling pathways such as the Notch, Wnt, and nuclear factor-κB signaling pathways. In cancer, TLE1 has oncogenic functions in lung cancer (Allen et al., 2006) and synovial sarcoma (Seo et al., 2011) in addition to tumor-suppressing activity in hematologic malignancies (Fraga et al., 2008). In an *in vitro* study of a LUAD cell line, TLE1 was shown to potentiate the epithelial-to-mesenchymal transition in part through the suppression of the tumor suppressor gene E-cadherin. It also provides a mechanism underlying the oncogenic activity of TLE1 in lung cancer (Yao et al., 2014). Collectively, these findings support the hypothesis that the prognosis-related genes discovered by CWx are highly likely to be useful as prognostic biomarkers for LUAD given further experimental and clinical validation.

In summary, we have developed a novel prognosis-related feature selection framework called CWx. Intriguingly, the top 100 gene set identified by the algorithm was related to the Wnt signaling pathway, which has been reported to be associated with the prognosis of LUAD (Xu et al., 2017; Han et al., 2018). Further experimental and clinical validation is required to demonstrate the prognostic potential of the top 100 genes identified by CWx.

## Author Contributions

BS, SP, YK, and KeK designed the study. BS and SP developed the CWx algorithm. KeK and KiK performed bioinformatic analyses. JH, HA, SC, KiK, and Y-HA contributed to the dataset for this study. BS, SP, JH, YK, and KeK wrote the manuscript. All authors read and approved the final manuscript.

## Funding

This study was supported by a grant from the National R&D Program for Cancer Control, Ministry of Health & Welfare, Republic of Korea (1720100).

## Conflict of Interest Statement

Authors BS, SP, and KiK were employed by company Deargen Inc. The remaining authors declare that the research was conducted in the absence of any commercial or financial relationships that could be construed as a potential conflict of interest.
