# Supplementary material for: Cascaded Wx: A Novel Prognosis-Related Feature Selection Framework in Human Lung Adenocarcinoma Transcriptomes
Source: Front Genet. 2019 Jul 19;10:662. doi: 10.3389/fgene.2019.00662 (PMC6658675; doi:10.3389/fgene.2019.00662)
Supplement: Supplementary file 1 [file DataSheet_1.pdf]

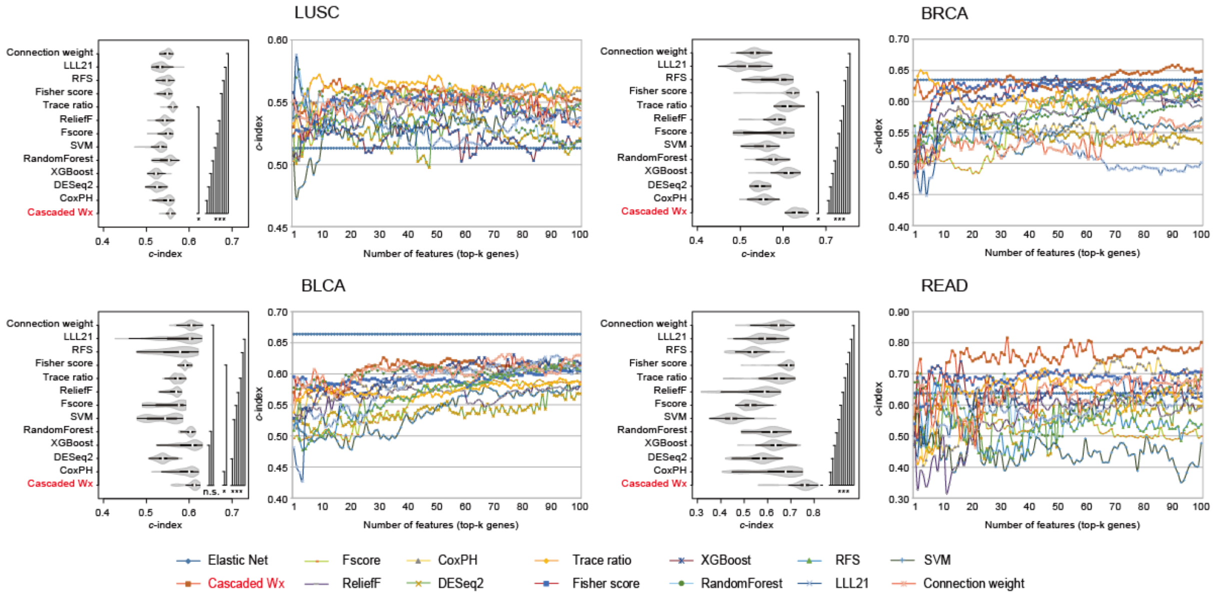

Figure S1. Comparison of feature selection algorithms with cumulative top-k genes

Violin plot shows *c*-indexes of the top genes (cumulative from 1 to 100, n = 100) identified by each algorithm (left panel). White circles indicate the medians; box limits inside the polygons indicate the 25th and 75th percentiles as determined by R software; whiskers extend 1.5 times the interquartile range from the 25th and 75th percentiles; polygons represent density estimates of data and extend to extreme values. Asterisks (**P* < 0.05; ****P* < 0.001; n.s., not significant) indicate results of one-way ANOVA (*P* value < 0.0001) with post hoc test (pairwise t-test with Bonferroni-Holm correction). The x-axis and y-axis indicate the number of cumulative top genes and *c*-index, respectively (right panel).

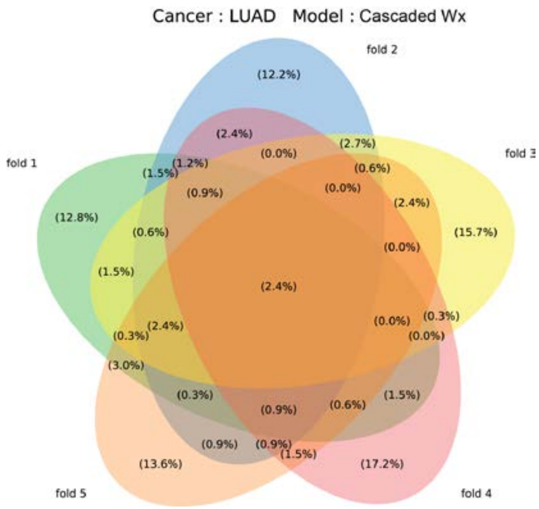
Cancer : LUAD Model : Cascaded Wx
fold 1
fold 2
fold 3
fold 4
fold 5
(12.2%)
(2.4%)
(0.0%)
(2.7%)
(1.5%)
(1.2%)
(0.6%)
(0.9%)
(0.0%)
(2.4%)
(12.8%)
(0.6%)
(15.7%)
(0.0%)
(1.5%)
(2.4%)
(2.4%)
(0.0%)
(0.3%)
(0.3%)
(0.0%)
(3.0%)
(0.3%)
(1.5%)
(0.9%)
(0.6%)
(0.9%)
(0.9%)
(1.5%)
(13.6%)
(17.2%)

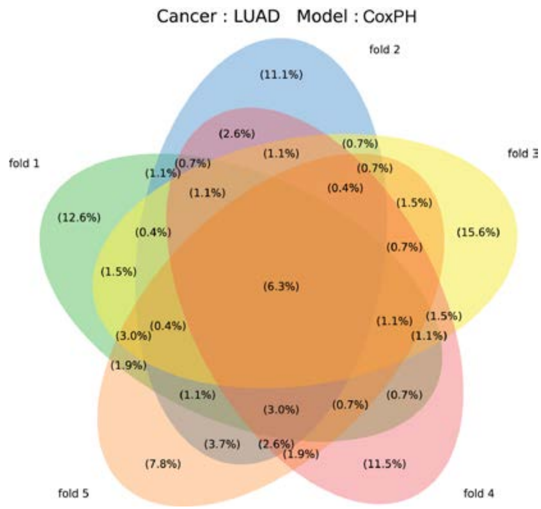
Cancer : LUAD Model : CoxPH
fold 1
fold 2
fold 3
fold 4
fold 5
(11.1%)
(2.6%)
(1.1%)
(0.7%)
(1.1%)
(0.7%)
(0.7%)
(1.1%)
(0.4%)
(1.5%)
(12.6%)
(0.4%)
(15.6%)
(0.7%)
(1.5%)
(6.3%)
(0.4%)
(1.1%)
(1.5%)
(3.0%)
(1.1%)
(1.9%)
(1.1%)
(0.7%)
(3.0%)
(0.7%)
(3.7%)
(2.6%)
(1.9%)
(7.8%)
(11.5%)

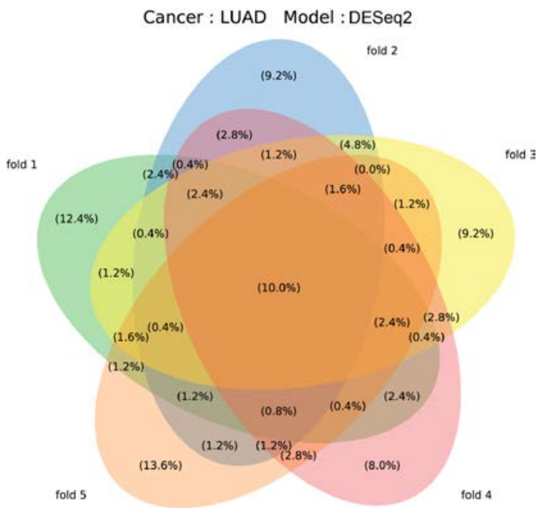
Cancer : LUAD Model : DESeq2
fold 1
fold 2
fold 3
fold 4
fold 5
(9.2%)
(2.8%)
(1.2%)
(4.8%)
(2.4%)
(0.4%)
(0.0%)
(2.4%)
(1.6%)
(1.2%)
(12.4%)
(0.4%)
(9.2%)
(0.4%)
(1.2%)
(10.0%)
(0.4%)
(2.4%)
(2.8%)
(1.6%)
(0.4%)
(1.2%)
(1.2%)
(2.4%)
(0.8%)
(0.4%)
(1.2%)
(1.2%)
(2.8%)
(13.6%)
(8.0%)

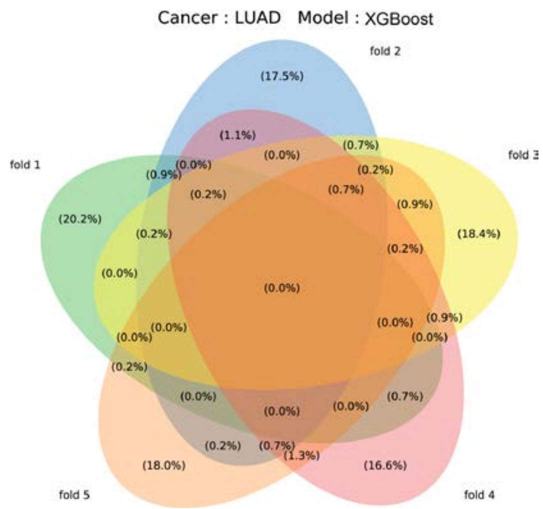
Cancer : LUAD Model : XGBoost
fold 1
fold 2
fold 3
fold 4
fold 5
(17.5%)
(1.1%)
(0.0%)
(0.7%)
(0.9%)
(0.0%)
(0.2%)
(0.2%)
(0.7%)
(0.9%)
(20.2%)
(0.2%)
(18.4%)
(0.2%)
(0.0%)
(0.0%)
(0.0%)
(0.0%)
(0.9%)
(0.0%)
(0.0%)
(0.2%)
(0.0%)
(0.7%)
(0.0%)
(0.0%)
(0.2%)
(0.7%)
(1.3%)
(18.0%)
(16.6%)

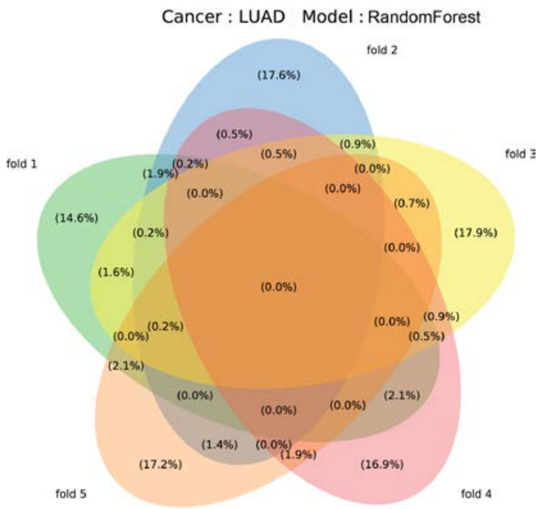
Cancer : LUAD Model : RandomForest
fold 1
fold 2
fold 3
fold 4
fold 5
(17.6%)
(0.5%)
(0.5%)
(0.9%)
(1.9%)
(0.2%)
(0.0%)
(0.0%)
(0.0%)
(0.7%)
(14.6%)
(0.2%)
(17.9%)
(0.0%)
(1.6%)
(0.0%)
(0.2%)
(0.0%)
(0.9%)
(0.0%)
(0.5%)
(2.1%)
(0.0%)
(2.1%)
(0.0%)
(0.0%)
(1.4%)
(0.0%)
(1.9%)
(17.2%)
(16.9%)

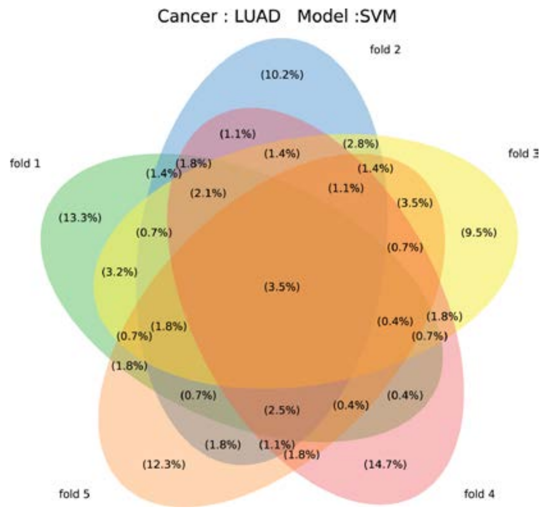
Cancer : LUAD Model :SVM
fold 1
fold 2
fold 3
fold 4
fold 5
(10.2%)
(1.1%)
(1.4%)
(2.8%)
(1.4%)
(1.8%)
(1.4%)
(2.1%)
(1.1%)
(3.5%)
(13.3%)
(0.7%)
(9.5%)
(0.7%)
(3.2%)
(3.5%)
(1.8%)
(0.4%)
(1.8%)
(0.7%)
(0.7%)
(1.8%)
(0.7%)
(0.4%)
(2.5%)
(0.4%)
(1.8%)
(1.1%)
(1.8%)
(12.3%)
(14.7%)

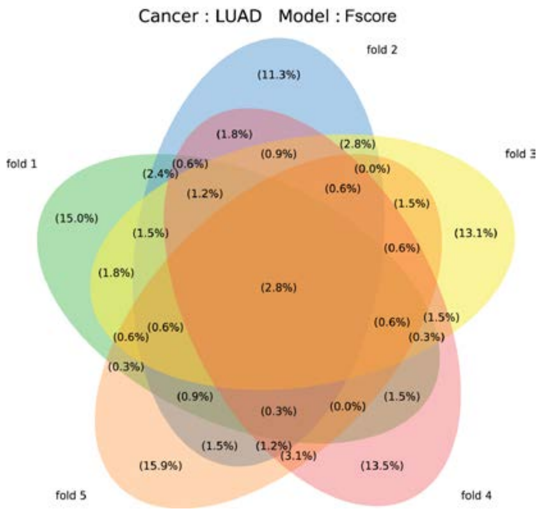
Cancer : LUAD Model : Fscore
fold 1
fold 2
fold 3
fold 4
fold 5
(11.3%)
(1.8%)
(0.9%)
(2.8%)
(2.4%)
(0.6%)
(0.0%)
(1.2%)
(0.6%)
(1.5%)
(15.0%)
(1.5%)
(13.1%)
(0.6%)
(1.8%)
(2.8%)
(0.6%)
(0.6%)
(1.5%)
(0.6%)
(0.3%)
(0.3%)
(0.9%)
(0.3%)
(0.0%)
(1.5%)
(1.5%)
(1.2%)
(3.1%)
(15.9%)
(13.5%)

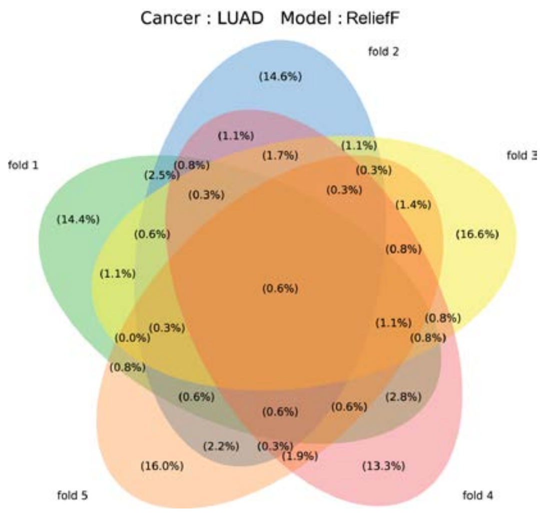
Cancer : LUAD Model : ReliefF
fold 1
fold 2
fold 3
fold 4
fold 5
(14.6%)
(1.1%)
(1.7%)
(1.1%)
(2.5%)
(0.8%)
(0.3%)
(0.3%)
(0.3%)
(1.4%)
(14.4%)
(0.6%)
(16.6%)
(0.8%)
(1.1%)
(0.6%)
(0.3%)
(1.1%)
(0.8%)
(0.0%)
(0.8%)
(0.8%)
(0.6%)
(0.6%)
(0.6%)
(2.8%)
(2.2%)
(0.3%)
(1.9%)
(16.0%)
(13.3%)

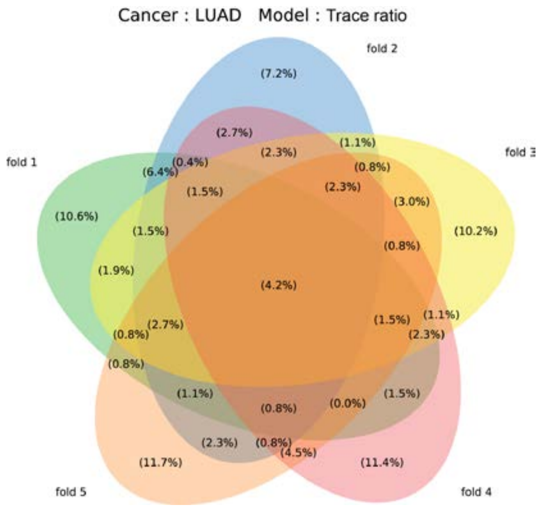
Cancer : LUAD Model : Trace ratio
fold 1
fold 2
fold 3
fold 4
fold 5
(7.2%)
(2.7%)
(2.3%)
(1.1%)
(6.4%)
(0.4%)
(0.8%)
(1.5%)
(2.3%)
(3.0%)
(10.6%)
(1.5%)
(10.2%)
(0.8%)
(1.9%)
(4.2%)
(2.7%)
(1.5%)
(1.1%)
(0.8%)
(2.3%)
(0.8%)
(1.1%)
(0.8%)
(0.0%)
(1.5%)
(2.3%)
(0.8%)
(4.5%)
(11.7%)
(11.4%)

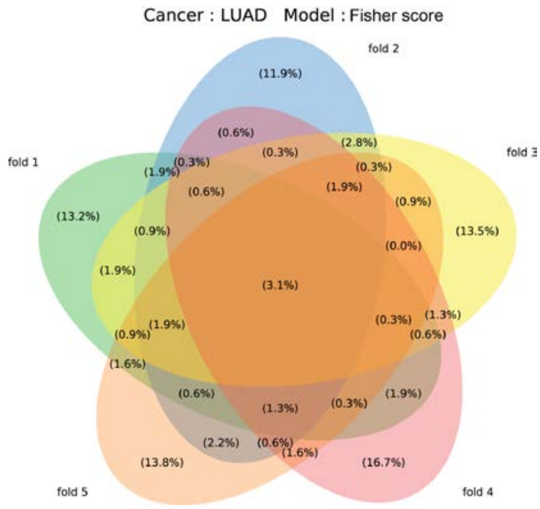
Cancer : LUAD Model : Fisher score
fold 1
fold 2
fold 3
fold 4
fold 5
(11.9%)
(0.6%)
(0.3%)
(2.8%)
(1.9%)
(0.3%)
(0.3%)
(0.6%)
(1.9%)
(0.9%)
(13.2%)
(0.9%)
(13.5%)
(0.0%)
(1.9%)
(3.1%)
(1.9%)
(0.3%)
(1.3%)
(0.9%)
(0.6%)
(1.6%)
(0.6%)
(1.3%)
(0.3%)
(1.9%)
(2.2%)
(0.6%)
(1.6%)
(13.8%)
(16.7%)

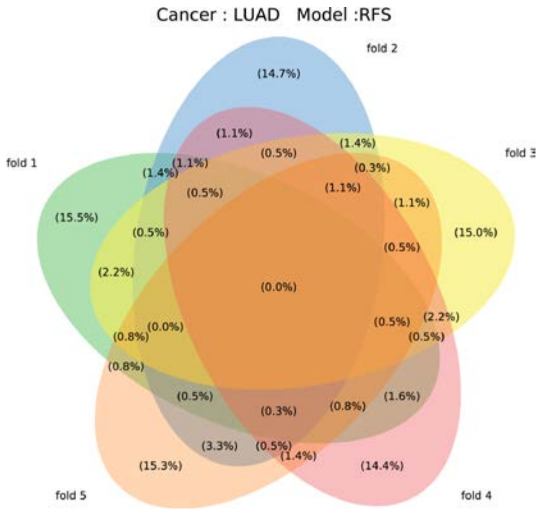
Cancer : LUAD Model :RFS
fold 1
fold 2
fold 3
fold 4
fold 5
(14.7%)
(1.1%)
(0.5%)
(1.4%)
(1.4%)
(1.1%)
(0.3%)
(0.5%)
(1.1%)
(1.1%)
(15.5%)
(0.5%)
(15.0%)
(0.5%)
(2.2%)
(0.0%)
(0.0%)
(0.5%)
(2.2%)
(0.8%)
(0.5%)
(0.8%)
(0.5%)
(0.3%)
(0.8%)
(1.6%)
(3.3%)
(0.5%)
(1.4%)
(15.3%)
(14.4%)

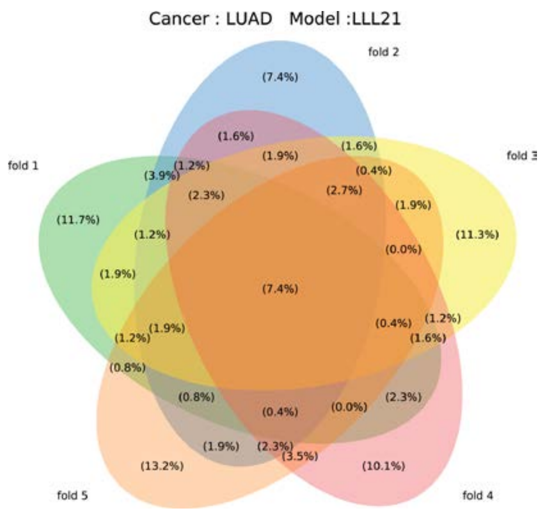
Cancer : LUAD Model :LLL21
fold 1
fold 2
fold 3
fold 4
fold 5
(7.4%)
(1.6%)
(1.9%)
(1.6%)
(3.9%)
(1.2%)
(0.4%)
(2.3%)
(2.7%)
(1.9%)
(11.7%)
(1.2%)
(11.3%)
(0.0%)
(1.9%)
(7.4%)
(1.9%)
(0.4%)
(1.2%)
(1.2%)
(1.6%)
(0.8%)
(0.8%)
(0.4%)
(0.0%)
(2.3%)
(1.9%)
(2.3%)
(3.5%)
(13.2%)
(10.1%)

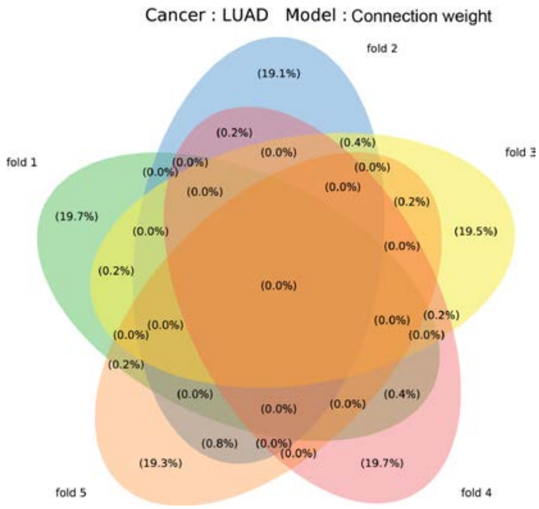

Figure S2. Intersection of genes between five folds (groups). Venn diagrams show the intersection of selected genes in the 5-fold cross-validation using feature-selection algorithms.
